# Polymers and Bioactive Compounds with a Macrophage Modulation Effect for the Rational Design of Hydrogels for Skin Regeneration

**DOI:** 10.3390/pharmaceutics15061655

**Published:** 2023-06-05

**Authors:** Mirna L. Sánchez, Hugo Valdez, Micaela Conde, Pamela Viaña-Mendieta, Aldo R. Boccaccini

**Affiliations:** 1 Laboratorio de Farmacología Molecular, Departamento de Ciencia y Tecnología, Universidad Nacional de Quilmes, Bernal B1876, Argentina; 2 Institute of Biomaterials, Department of Materials Science and Engineering, University of Erlangen-Nuremberg, Cauerstrasse 6, 91058 Erlangen, Germany; 3 Laboratorio de Microbiología Celular e Inmunomecanismos, CINDEFI|Centro de Investigación y Desarrollo en Fermentaciones Industriales Facultad de Ciencias Exactas, La Plata B1900AJL, Argentina; 4 Tecnologico de Monterrey, Instituto para la Investigación en Obesidad, Monterrey 64849, Mexico

**Keywords:** biomaterials, inmunomodulation, macrophages M1–M2, polymers, wound-healing

## Abstract

The development of biomaterial platforms for dispensing reagents of interest such as antioxidants, growth factors or antibiotics based on functional hydrogels represents a biotechnological solution for many challenges that the biomedicine field is facing. In this context, in situ dosing of therapeutic components for dermatological injuries such as diabetic foot ulcers is a relatively novel strategy to improve the wound healing process. Hydrogels have shown more comfort for the treatment of wounds due to their smooth surface and moisture, as well as their structural affinity with tissues in comparison to hyperbaric oxygen therapy, ultrasound, and electromagnetic therapies, negative pressure wound therapy or skin grafts. Macrophages, one of the most important cells of the innate immune system, have been described as the key not only in relation to the host immune defense, but also in the progress of wound healing. Macrophage dysfunction in chronic wounds of diabetic patients leads to a perpetuating inflammatory environment and impairs tissue repair. Modulating the macrophage phenotype from pro-inflammatory (M1) to anti-inflammatory (M2) could be a strategy for helping to improve chronic wound healing. In this regard, a new paradigm is found in the development of advanced biomaterials capable of inducing in situ macrophage polarization to offer an approach to wound care. Such an approach opens a new direction for the development of multifunctional materials in regenerative medicine. This paper surveys emerging hydrogel materials and bioactive compounds being investigated to induce the immunomodulation of macrophages. We propose four potential functional biomaterials for wound healing applications based on novel biomaterial/bioactive compound combination that are expected to show synergistic beneficial outcomes for the local differentiation of macrophages (M1–M2) as a therapeutic strategy for chronic wound healing improvement.

## 1. Introduction 

Wound healing is an exceedingly complex and finely regulated process, essential to preserve healthy conditions. Acute wounds usually proceed through an organized and appropriate repair process that eventually results in the regaining of skin structural integrity, following trauma, burns, or surgery. However, chronic wounds have persistent inflammation due to a disruption of the wound healing cycle as a result of impaired angiogenesis, innervation, or cellular migration, among other reasons [1,2]. There are many illnesses which can cause these types of complications. Diabetes mellitus is one of the most well known in relation to complex wound healing processes, and high incidence of complications relate to this. Failure to heal, such as diabetic foot ulcers, can even lead to limb amputation in the most severe cases [3].

In recent years, knowledge of the different factors involved in the repair of wounds has progressed enormously. Macrophages, one the most important cells of the innate immune system, have been described as key factors not only in the host immune defense, but also in the resolution of wound healing. There has been increasing evidence showing macrophage dysfunction in chronic wounds of diabetic patients, leading to a perpetuating inflammatory environment and preventing reparative cell infiltration [4]. Moreover, improperly acting macrophages lead to local environment malfunctioning, inducing the accumulation of inflammatory cytokines and degraded extracellular matrix [5,6]. 

Macrophages are highly plastic cells capable of rapidly adopting a range of phenotypes depending on the environment to ensure homeostasis, the clearance of debris and proper healing [7,8,9,10]. They have been frequently classified into two extreme phenotypes in response to cytokine cues: classically activated or pro-inflammatory macrophages (M1), and alternatively activated or anti-inflammatory macrophages (M2). M1s are activated in response to interferon-γ (IFN-γ) and lipopolysaccharide (LPS). In a polarized response, they produce abundant pro-inflammatory cytokines such as tumor necrosis factor α (TNF-α), and pro-inflammatory mediators such as nitric oxide (NO). While M2s are stimulated by IL-4 or IL-13 and secrete transforming growth factor (TGF-b), IL-10, and other growth factors, such as platelet-derived growth factor (PDGF), resistin such as molecule α (Relmα), epidermal growth factor, and vascular endothelial growth factor-a (VEGFA), leads to tissue repair and inflammation resolution based on different pathways [8,11,12,13,14].

Modulating the macrophage polarization could be a strategy to improve chronic wound closure. Wound healing is indeed a complex process, and advancements in this area can have a significant impact on healthcare. The speed of wound closure is one important aspect of wound healing, but there are many other factors to consider as well, such as reducing the risk of infection, minimizing scarring, and promoting proper tissue regeneration. Advancements in wound healing should improve patient outcomes and quality of life by promoting faster and more effective healing, reducing the risk of complications, and minimizing scarring and other long-term effects of wounds. 

Recent results have shown that external intervention could lead to better wound closure times and tissue regeneration [15,16,17,18,19,20,21,22,23]. In this regard, a new paradigm is emerging: the development of advanced biomaterials in combination with biological active molecules or drugs capable of inducing macrophage polarization, and thus the acceleration of wound healing [24,25].

The term ‘immunobioengineering’ is used to describe efforts by immunologists and bioengineers to design biomaterials as delivery vehicles targeting the immune response [26]. In this context, the engineering of materials that can modulate the immune system is an emerging field that is developing alongside immunology. An interesting general overview underlying the steps of the host immune response upon exposure to biomaterials has been reported by Bu et al. [27]. 

In particular, considering novel materials for in situ immunomodulation, these advances contribute not only to the wound healing field, but also to the progress of materials engineering in general.

Strategies to enhance, suppress, or qualitatively shape the immune response are of importance for diverse biomedical applications. However, the intricate cellular and molecular signals regulating the immune system are extremely complicated to manipulate. To meet this challenge, biomaterials are being developed intending to control how and when some specific immune cells are stimulated in vivo. 

The efficiency and biocompatibility of some therapeutic drugs can be significantly improved through encapsulation within biomaterials. This approach provides protection from enzymatic degradation and improves its pharmacokinetics [28]. Some biomaterials have been designed to deliver immunomodulatory agents to the injury site, for example, in order to improve the wound healing process [24,25,29,30,31]. Most of them are hydrogels; their high water content to emulate hydrated physiological environments, versatility, fine tunability, and physical properties make them promising biomaterial candidates for a variety of biomedical applications [32]. Different crosslinking degrees, elasticity modulus, and pore sizes can be achieved through the optimization of the hydrogel synthesis. Besides the physical characteristics, biochemical properties provide a wide opportunity to design and customize hydrogels for specific applications. Therefore, optimizing physicochemical properties and understanding the relationship between hydrogels (as carriers) and bioactive compounds will contribute to develop novel ‘immunobioengineering systems.

Thus, the goal of this paper is to discuss the actual approaches to treat chronic wounds, based on literature results which report hydrogels loaded with bioactive compounds characterized by their macrophage modulation activity. 

It is important to remark that the present paper will focus only on available literature which considers only the biochemical role of the hydrogel components to achieve an immunomodulatory activity. There are other factors which contribute to “better” hydrogels for this purpose. For example, topography, porosity, stiffness, electrical properties, the degree of degradation, etc. [27]. Nevertheless, our discussion will be specifically confined to biomaterials and molecular signals that regulate the immune response by M1/M2 polarization based on biochemical effects. 

The rational design of immunomodulating biomaterials is complex and there is plenty of research to be conducted on developing customized materials for this purpose. As a contribution to this evolving field, in this paper, we analyzed the relevant literature and proposed hydrogel/immunomodulator combinations as innovative biomedical platforms for specific applications in chronic wound treatments.

## 2. Macrophages 

### 2.1. Macrophage Polarization Role in Wound Healing

This section will briefly describe how the immune system acts in the wound healing process, focusing on the macrophage role. Wound healing involves extensive and complex communication between different cells of the immune system such as neutrophils, monocytes, and macrophages, among others. Traditionally, the wound healing process is characterized by four phases that occur in a temporal sequence, but are partially overlapping: homeostasis, inflammation, proliferation, and remodeling [1,33]. 

Neutrophils are the first immune cells to respond to an injury, and they play a critical role in the early stages of wound healing by phagocytosing bacteria and debris. However, excessive neutrophil infiltration and activation can also cause tissue damage and impair wound healing. In chronic wounds, neutrophil infiltration can persist, leading to a sustained inflammatory response that can delay healing [34]. 

Lymphocytes, specifically T cells and B cells, also play a role in the wound healing process by promoting angiogenesis, tissue remodeling, and collagen synthesis. T cells can produce cytokines that stimulate fibroblasts and endothelial cells, while B cells can produce antibodies that promote wound healing [35]. 

However, the role of neutrophils and lymphocytes in chronic wounds is highly complex and context-dependent, and their contributions to the healing process are not as well understood as that of macrophages. Therefore, macrophages are the focus of the present discussion, because the manipulation of macrophage polarization represents an opportunity for developing new technologies based on biomaterials, to contribute to tackling the problem of chronic wound healing.

Macrophage polarization plays a key role in leading the phases during the wound healing response. During early wound healing, macrophages that invade the tissue exhibit an inflammatory phenotype (M1) [36]. These activated macrophages act in pathogen phagocytosis, destroy and remove damaged cells, including spent neutrophils, while recruiting additional inflammatory cells. However, macrophage populations are dynamic, and their phenotype is plastic, being able to change completely. In the following phase, the classically activated M1 phenotype gradually skews toward a subset of the alternatively activated M2 phenotype, such as M2a, M2b, and M2c, that down-regulates inflammation and promotes repair and regeneration. M2s are induced by a mix of stimuli, such as the phagocytosis of apoptotic spent neutrophils, IL-4 or IL-13, and produce anti-inflammatory cytokines such as TGF-b and IL-10 [37,38,39,40]. In diabetic wounds, prolonged inflammation can result in fibrotic wound healing, and dysregulation of the immune response during wound healing leads to the emergence of chronic wounds [41,42,43]. In such wounds, the M1 to M2 macrophage switch fails. Unlike in acute wounds, macrophages are unable to phagocytose neutrophils. This effect leads to the recruitment of more macrophages and an increase in inflammation.

During the re-epithelialization stage, macrophages secrete vascular endothelial growth factors and promote the proliferation of endothelial cells, skeletal myoblasts, and fibroblasts. This phase is followed by angiogenesis, myotube formation, and collagen production. The final phase involves collagen remodeling for the formation of new epithelium and scar tissue. 

As mentioned earlier, macrophage polarization plays an important role throughout the wound healing process and a dysfunction in their performance can lead to the dysregulation of normal wound healing [41,44]. Diabetic patients with wounds, such as the case of diabetic foot ulcers, usually suffer from non-healing with inflammation and microbial infection. In these cases, the wound healing response is altered due to dysregulation in macrophage function and disruption of the transition of M1 macrophages to M2 macrophages, which may eventually lead to a delay in wound closure. The study with a selective depletion of macrophages during the wound healing process led to delayed re-epithelialization, reduced collagen deposition, impaired angiogenesis, and decreased cell proliferation. These impairments were associated with an increased expression of TNF and reduced expression of VEGF and TGF-1, indicating that macrophages help regulate the cytokine environment during wound healing. The authors speculate that the dysfunction of macrophages may contribute to the development of chronic wounds [45]. Although the importance of the inflammatory phase has been demonstrated, wherein macrophages in their pro-inflammatory phase were observed to promote vessel sprouting and growth of blood vessels in the wound tissue, a prolonged inflammatory phase plays a detrimental role in wound closure [8,46,47]. In this type of wound, a difficult resolution of polarization is observed, the polarization to the M2 phenotype is stopped, and the anti-inflammatory state and re-epithelization do not occur. 

### 2.2. Novel Approaches in Immunomodulation

**Figure 1:**
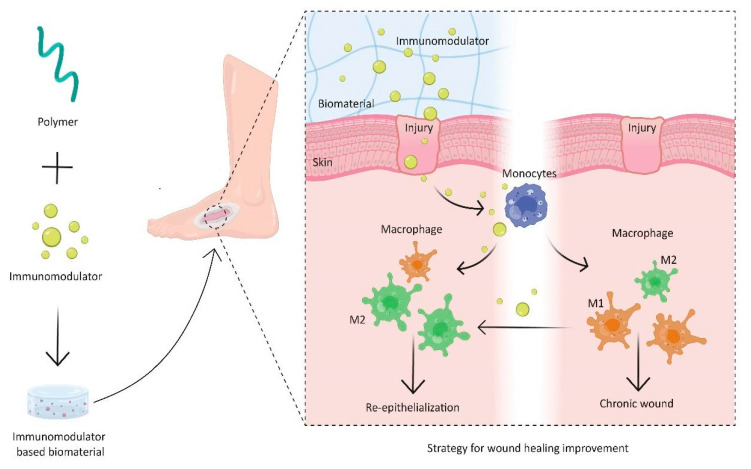
Polymers and bioactive compounds in hydrogels for skin regeneration promoted by macrophage polarization from M1 to M2.

Different options have been proposed for the treatment of chronic wounds over the years, including the use of biologic agents, bioactive materials and cell therapies [7,48,49]. Many current approaches focus on the attenuation of macrophages M1 and the promotion of their transformation into the M2 phenotype. This strategy is represented in Figure 1. 

In addition to immunomodulators, the endogenous properties of the gels themselves can also modulate macrophages, such as hydrogels composed of hyaluronic acid, low-molecular-weight pro-M1, and high-molecular-weight pro-M2.

M2 macrophages can be obtained in vitro in the presence of IL-4, IL-10, or IL-13, but this stimulation does not work fine in an acute wound to switch from M1 to M2 phenotypes. For this reason, new approaches have been proposed to favor the modulation of the macrophage phenotype that favors wound repair [50,51]. 

For example, Fu and colleagues investigated quercetin effects on wound healing in diabetic rats [52]. Quercetin is one of the most abundant flavonoids, it is found in the leaves and fruits of various plants. It is a versatile molecule with many pharmacological properties such as anti-cancer, anti-oxidation, anti-fibrosis, and anti-inflammation effects [53,54]. For this reason, quercetin is being deeply investigated in different biomedical conditions. According to the researchers, quercetin reduced the expression of pro-inflammatory factors and increased the expression of anti-inflammatory factors via modulating the macrophage polarization switching from the M1 to M2 phenotype, which resulted in the acceleration of wound closure [34]. He and colleagues investigated the potential use of interleukin (IL)-33. IL-33 is a member of the IL-1 cytokine family that binds to receptor ST2. This study showed that exogenous IL-33 application promotes M2 macrophage polarization in diabetic mice [34]. Subsequently, TGF-β secretion through the amplified M2 macrophages significantly augmented the proliferation of fibroblasts and the production of ECM-associated collagens. However, little is known about the mechanism of action of IL-33 [22]. 

Following the same approach based on macrophage polarization, Yu and colleagues investigated the effect of HG (high glucose) plus insulin on the macrophage phenotype polarization using a human monocytic THP-1 cell and diabetic rat model [23]. It is well known that the disruption of the insulin signaling pathway is one of the most distinctive pathological changes of type II diabetes [15,23]. In an in vitro study, researchers proved that HG plus insulin promotes the macrophage phenotype transition from M1 to M2, and attenuates inflammatory mediator secretion. Such results confirmed that both PI3K-Akt-Rac1 and PPAR-γ signaling pathways are involved in the insulin-induced macrophage phenotype switch and anti-inflammatory effect [23]. 

Similarly, Liu and colleagues showed that M2 polarization could be promoted and M1 polarization inhibited by activating the PTEN/AKT signaling pathway [55]. This could be achieved by means of melatonin (MT)-pre-treated MSC-derived exosomes (MT-Exo). Exosomes are lipid bilayer particles which are naturally secreted from the cells. They are considered nanocarriers because they can transport different cargoes, such as micro-RNAs (miRNAs), proteins, etc [56]. Currently, studies have revealed that MSC-derived extracellular vesicles result in phenotypic change and modification in target organs. These phenotypic alterations are triggered by the attenuation of oxidative stress in recipient cells, prevention of apoptosis in target cells, and other mechanisms [57,58]. In addition to exosomes, melatonin is a hormone that suppresses lipopolysaccharide (LPS)-induced pro-inflammatory factors released in macrophages. Many actions of melatonin are mediated through interaction with the melatonin receptors 1 and 2, which are G-protein-coupled membrane receptors found in several cell types [59,60,61].

Other approaches refer to the regulation of the AMPK/mTOR signaling pathway in order to inhibit the activation of the NLRP3 inflammasome. This is due to the emerging evidence indicating that down-regulation of the NLRP3 inflammasome and cleaved IL-1β inhibit inflammation, improving wound healing [62,63]. For this reason, Qing and colleagues decided to investigate the effects of metformin on wound healing [64]. Metformin (dimethylbiguanide) is a synthetic guanidine derivative that lowers glucose levels by inhibiting hepatic glucose production [57,65]. Furthermore, metformin inhibits the expression of pro-inflammatory cytokines and protects against oxidative damage [66,67]. It was demonstrated that metformin could inhibit the activation of the NLRP3 inflammasome by regulating the AMPK/mTOR singularization pathway, thus achieving the polarization of M2 macrophages and accelerating the wound healing process. 

Meanwhile, Dai and colleagues proposed that the use of rapamycin could attenuate NLRP3 inflammasome activation in macrophages by inhibiting mTOR phosphorylation [68]. As is known, mTOR acts as a regulator of the levels of pro- and anti-inflammatory cytokines [68]. On the other hand, rapamycin, an mTOR inhibitor, is a potent immunosuppressive drug that promotes wound healing by enhancing autophagy [69]. Therefore, rapamycin could be a possible therapeutic option for the inflammatory response in impaired wound healing, but further studies are needed to determine its therapeutic potential in vivo [70].

Table 1 presents a summary of the various strategies employed to immunomodulate macrophages in mouse models of chronic wounds using bioactive compounds as immunomodulators. The table also includes information on the implicated pathway alterations and their effects on the wound healing process. This is an incipient field, and we expect that the information in the table will be the kick-off for starting a search for analogue biomaterials capable of inducing the same functional effect on macrophages. 

Analyzing the data reported, it can be observed that many bioactive compounds share common action pathways, with the control of the inflammasome activation pathways being of relevance. 

Hassanshahi et al. reviewed and described an apparent intricate relationship between inflammasome activation and recruitment, and macrophage polarization during wound healing [72]. Therefore, new effects that will have action on the inflammasome pathways may be of interest to evaluate the progress of different wound repair processes.

## 3. Hydrogels in Immunomodulation Strategies

Hydrogels are interesting biomaterials for the controlled release of bioactive molecules (in particular pharmaceutical proteins) and cell encapsulation. The biodegradable hydrogel structure disintegrates into nontoxic substances to induce an excellent biocompatibility of the gel. Chemical cross-linking is the highly resourceful method for the formation of hydrogels, having the required mechanical strength. Often, cross-linkers used in hydrogel preparation should be extracted from the hydrogels before use due to their reported toxicity. Physically cross-linked methods for the preparation of hydrogels are the alternative solution of cross-linker toxicity and are of huge interest for labile bioactive substance and cell encapsulation and entrapment, especially when the hydrogel development is conducted in the absence of organic solvents and under mild conditions [73]. 

Drug loading procedures can be performed at two different times of the hydrogel synthesis: at the beginning by mixing the drug with the other reagents, or at the end, after the hydrogel has been produced [74].

In situ loading methods are suitable for hydrophilic drugs and are based on dissolving the drug in water together with the polymer powder. The other technique is called post-loading and refers to dry hydrogel film immersion into a drug solution for a certain period of time. In both cases, after drug incorporation, the hydrogel is in a dried state and confers protection. In addition, cross-linkers are the essential factors in controlling the release of high- or low-molecular-weight therapeutic agents, and in most cases the degradable cross-linkers are preferred [75].

The inherent properties of hydrogels allow for the damaged tissue to heal by supporting a hydrated environment which has long been explored in wound management to aid in autolytic debridement. However, chronic non-healing wounds require added therapeutic features that can be achieved through the incorporation of biomolecules and supporting cells to promote faster and better healing outcomes. In recent decades, numerous hydrogels have been developed and modified to match the time scale for distinct stages of wound healing.

Hydrogels can be used to deliver bioactive molecules known to accelerate wound healing, or support and maximize the therapeutic potential of skin or stem cells to promote angiogenesis and re-epithelialization, as well as new extracellular matrix (ECM) production and maturation [76].

For example, Xiang et al. 2023 developed a hydrogel wound dressing loaded with melanin nanoparticles in a polysaccharide matrix (biguanide chitosan and oxidized β-glucan) for efficient healing of bacterially infected diabetic wounds. This hydrogel has a three-dimensional network structure that dramatically enhances cell proliferation, migration, and angiogenesis by creating favorable ecological conditions for cell survival. Melanin nanoparticles have a polyphenolic structure on their surface, giving them good ROS scavenging ability. The novelty of this work is that together with near-infrared (NIR) photothermal therapy, melanin nanoparticles can effectively kill bacteria and regulate the oxidative stress state, relieving the inflammatory response and promoting the transition of diabetic wounds from the inflammatory stage to the proliferative stage [77]. 

Related to hydrogels for immunomodulation, Table 2 summarizes biomaterials that themselves have the ability to promote macrophage polarization. As mentioned before, there are natural polymers with excellent effective properties in wound healing. One of the most used is hyaluronic acid (HA), a major glycosaminoglycan of the extracellular matrix. Its importance is given by the role it plays in cell adhesion, inflammation and the interaction between cells [78,79,80]. High molecular weight HA (HHA) has been reported to promote the change of macrophage phenotypes from M1 to M2 through several mechanisms [30]. One of them is the reduction in the expression of toll-like receptor 4 (TLR-4) in the cells stimulated with LPS through the inhibition of NF-kB activation, which may explain the promotion of the phenotype of macrophages M2. For these reasons, Liu and his colleagues used electro-spun thioether-grafted hyaluronic acid nanofibers for the modulation of macrophages and accelerating wound healing [29]. In vivo studies demonstrated that they promoted wound regeneration in a chronic wound model. Furthermore, these materials modulate the microenvironment of wound inflammation through ROS scavenging, regulation of macrophage phenotype, and change of chemoattractant gradients [81,82]. 

In the meantime, Shen and colleagues investigated the synthesis of a hydrogel composed of sulfated chitosan and type I collagen [25]. The study showed that the production of pro-inflammatory cytokines was reduced, while the production of anti-inflammatory cytokines increased because of the attenuation of M1 macrophages. Similarly, Masry and colleagues have focused on an equine pericardial collagen matrix (sPCM) that is capable of scaffolding functionality during the course of wound healing [24,25,30,83]. This wound dressing promotes the change in macrophage polarization toward the M2 phenotype and improves wound healing by increasing collagen deposition and maturation. Previously, it was reported that patients with diabetic foot wounds were treated with equine pericardium. Briefly, 32 wounds in 22 patients were prospectively available for evaluation. On enrollment, the median wound size was 299 mm^2^. When the equine material was removed (mean, 2.9 weeks), 30 of the wounds (94%) had improved, with a median size of 115 mm^2^ and an average reduction in size of 44.3% (*p* < 0.0001). At 4 weeks, the average decrease in wound size was 52.3% (*p* < 0.0001). At 12 weeks, 15 wounds (47%) had healed. Thus the technology represents an interesting option to improve healing and accelerate wound closure time [83]. 

On the other hand, there are some polymers mentioned as zwitterionic capable of maintaining stable hydration through electrostatic interactions between zwitter ions and water molecules [31,84]. These were the ones that He and his colleagues made based on zwitterionic hydrogels (polySBMA) [31]. The researchers built on earlier investigations that stimulated cell proliferation, upregulated the secretion of growth factors, such as TGF-β1, and activated macrophage polarization from M1 to M2. With these results, it was assured that softer and more viscoelastic hydrogels promote the polarization of macrophages, providing more oxygen and nutrients in such a way that they promoted cell proliferation and migration [84].

The progress of biomaterials and tissue engineering has led to significant advances in wound healing, but there are still many challenges to be solved and improvements to be implemented. Further progress in unveiling new drugs and polymers that could promote the M1 to M2 polarization will help us design novel immunoregulatory hydrogels. In this context, well-designed and fabricated materials that can promote inflammatory cell infiltration and the secretion of anti-inflammatory cytokines will promote tissue regeneration. 

The correlation between biomaterials and the immune response reported in the literature has prompted us to suggest new approaches to create microenvironments that promote wound healing, as discussed in the next sections.

## 4. Biomaterials and Macrophage Immunomodulation: A New Perspective for Wound Healing 

Wound healing is a complex process involving the sequential activation of local and systemic cells. In addition, it requires enzymatic pathways for the repair and recovery of injured tissue [85]. 

The inflammatory phase begins at the onset of the injury and typically lasts for around 2–3 days, although in some cases it can last up to 5 days. During this phase, white blood cells, such as neutrophils and macrophages, are recruited to the wound site.

The proliferative phase can last up to 21 days and is characterized by the migration of fibroblasts and other cells to the wound site. These cells produce substances, such as collagen [86], that are necessary for the formation of new tissue and the closure of the wound. Interruptions in any of the phases of wound healing can result in poor healing. For example, if the inflammatory phase is disrupted, the wound may be more susceptible to infection. Similarly, if the proliferative phase is disrupted, there may be a delay in the formation of new tissue and the closure of the wound [87]. 

In chronic wounds, there is often a persistent pro-inflammatory environment that can lead to an overabundance of M1 macrophages and a delay in the transition to the M2 phenotype. This imbalance can impair the healing process and lead to the formation of non-healing or slow-healing chronic wounds. Therefore, promoting a shift toward M2 macrophage polarization in chronic wounds appears as a promising therapeutic strategy to accelerate the healing process [1]. 

The idea of proposing a synergy between a biomaterial with immunomodulatory properties and a molecule with the same properties is innovative and challenging.

Previous reports have proposed biomaterials that provided properties such as balance or the support of molecules that themselves exerted an immunomodulatory action on macrophages. However, a current tendency in biomaterials research is that the biomaterial (carrier) itself could exert an immunomodulatory action as well. 

In order to consider the improvement of the wound healing process and propose suitable combinations of biomaterials and bioactive compounds, Table 3 summaries several materials and molecules which have been proposed in the literature, giving an overview on both biomaterials and bioactive compounds regarding their capacity to induce the differentiation of macrophages, either inhibiting M1 (shown as M1−), promoting M2 (shown as M2+) or showing an additive effect (shown as M1−− or M2++), including also their associated wound closure times studied in mouse animal models.

For example, equine pericardial collagen and hyaluronic acid can achieve wound closure in a short period of time within the range of thirteen to fifteen days [23], followed by sulfated chitosan, which is in the range of sixteen to eighteen days, whereas sulfobetaine methacrylate takes between nineteen and twenty-one days to achieve the closure of a wound. Therefore, equine pericardial collagen and hyaluronic acid could be considered promising base materials that could promote wound closure in a short period of time. Thioether grafted hyaluronic is one of the promising materials in terms of macrophage modulation as well, promoting the M2 phenotype, and at the same time, inhibiting the M1 phenotype [29]. In contrast, other biomaterials could only promote the M2 phenotype [24,25,31,84]. 

Related to immunomodulators, melatonin and quercetin promote the M2 phenotype and inhibit the M1 phenotype. However, the metformin compound achieves faster wound closure within the range of ten to twelve days. IL-33 takes the longest period to promote wound closure, between sixteen and eighteen days. 

Table 3 gives an overview of which type of material and immunomodulator is the most suitable option to be chosen according to the analyzed literature and the potential synergistic effect that could be achieved.

The previous analysis, focusing on how each component of the materials and bioactive compounds proposed would affect the immunomodulatory activity of macrophages in the wound healing process, was the basis to propose four hydrogel/immunomodulator combinations as potential innovative biomedical hydrogels for chronic wounds treatment.

First, the combination of hyaluronic acid as a carrier material, along with melatonin and quercetin, has the potential to enhance wound repair through a synergistic effect on the immunomodulatory activity of macrophages. Hyaluronic acid acts as an excellent polymeric support that can enhance the regulatory capacity of quercetin over M1, while melatonin provides a strong stimulus over M2 profile induction. As mentioned before, quercetin is known to have anti-inflammatory properties and can reduce oxidative stress, while melatonin can promote angiogenesis and collagen synthesis. These properties can help accelerate the wound healing process. Furthermore, hyaluronic acid is a well-known biomaterial used in wound healing due to its ability to promote cell migration and proliferation. By combining hyaluronic acid with melatonin and quercetin, the resulting hydrogel has the potential to improve wound closure, reduce inflammation, and promote tissue regeneration. Therefore, the combination of hyaluronic acid, melatonin, and quercetin have promising results in wound repair due to their potential to enhance the immunomodulatory activity of macrophages and promote tissue regeneration. 

As a second proposal, a combination of collagen with metformin and quercetin can be suggested. Collagen is a well-known biomaterial used in wound healing due to its ability to promote cell proliferation, migration, and angiogenesis. It provides a scaffold for tissue repair and is essential for wound closure. Metformin is an anti-diabetic drug that has been found to have beneficial effects in wound healing. It has been shown to promote angiogenesis and reduce inflammation, which are essential for tissue repair. As previously mentioned, quercetin has anti-inflammatory properties and can reduce oxidative stress, which can help accelerate wound healing. The combination of these three components may provide a synergistic effect that enhances their individual properties and promotes wound repair. Collagen hydrogel provides selective and rapid wound closure times, and the action of metformin, combined with the effects of quercetin, could result in an excellent biomaterial for treating chronic wounds.

A third proposal would be the combination of sulfated chitosan with metformin and melatonin to promote wound repair through its immunomodulatory properties. Chitosan is a biopolymer that has been widely studied for its potential in wound healing. It has been shown to have antimicrobial, anti-inflammatory, and antioxidant properties. Sulfated chitosan is a modified form of chitosan that has improved biological properties, including enhanced wound healing activity. It has been shown to stimulate the migration and proliferation of cells involved in wound healing, such as fibroblasts and endothelial cells. Metformin, as previously mentioned, has beneficial effects in wound healing due to its anti-inflammatory and pro-angiogenic properties. Melatonin, on the other hand, has antioxidant and anti-inflammatory properties that can reduce tissue damage and promote tissue repair. The combination of these three components may provide a synergistic effect that enhances their individual properties and promotes wound repair. Sulfated chitosan with metformin and melatonin has the capability to regulate the proinflammatory pathways of M1 macrophages and stimulate the conversion of macrophages to the anti-inflammatory phenotype M2. This could help reduce inflammation and promote tissue regeneration, leading to faster wound healing.

Finally, our fourth proposal suggests combining sulfobetaine methacrylate with metformin and melatonin for wound healing. Although there is limited research on this specific combination, the individual properties of these components suggest that their combination could have potential benefits in promoting wound repair. Sulfobetaine methacrylate is a synthetic polymer known for its biocompatibility, hemocompatibility, and anti-inflammatory properties, which could aid in the wound healing process. Metformin and melatonin, as previously discussed, have an anti-inflammatory and immunomodulation capacity over macrophage phenotype, antioxidant, and pro-angiogenic properties that promote wound healing. Combining these three components may enhance their individual properties and have a synergistic effect on promoting wound repair.

Overall, all the proposed combinations promise interesting results in wound repair due to their immunomodulatory properties. However, further research is needed to determine the optimal combination of these components, and the appropriate dosage and duration of treatment to achieve the best outcomes in wound healing. Clinical trials are also necessary to validate the efficacy of this combination in treating chronic wounds.

**Figure 2:**
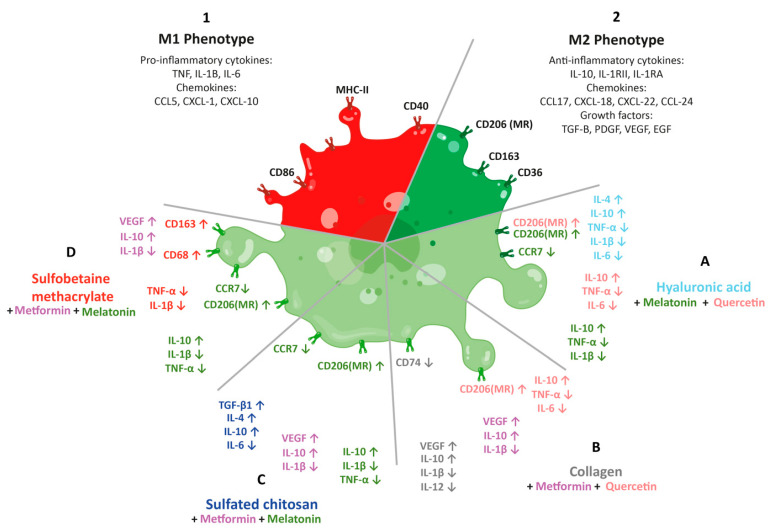
Macrophage phenotypes induced during the phase of the wound healing process and the phenotype expected through the modulation action of materials and immunomodulators. Repertoire of receptors, cytokines, and chemokines of the (1) M1 phenotype and (2) M2 phenotype. Repertoire of receptors, cytokines, and chemokines expected of (A) hyaluronic acid with melatonin and quercetin. (B) Collagen with metformin and quercetin. (C) Sulfate chitosan with metformin and melatonin. (D) Sulfobetaine methacrylate with metformin and melatonin. Up arrow: increment in the amount of this molecule. Down arrow: decrease in the amount of this molecule. In the same color are indicated bioactive molecule or biomaterial and its impact (increment o decrease) on the receptors, cytokines, and chemokines.

As mentioned previously, a critical analysis of the reported outcomes was carried out, leading to the selection of the proposed biomaterial combinations. This analysis involved determining how the main signaling molecules of the M1 and M2 pathways could be affected. The M1–M2 macrophage differentiation process implies complex pathways that are mostly already characterized. The developed survey focusing on how such pathways could be affected by the respective polymer–bioactive compound provides insights on how the differentiation process occurs, and most importantly, how it could be manipulated to obtain the desirable results. In Figure 2, the results of this analysis are shown, indicating how the polymer–bioactive compounds could modify the different signals implied in the M1–M2 polarization process.

The analysis provides useful information because the molecules analyzed are involved in other metabolic pathways as well. For example, quercetin acts in the down-regulation of the activity of NF-kB and IRF-5, reducing the synthesis and release of key inflammatory factors for the M1 pathway activation. The melatonin effect works by inhibiting the activation of the PI3K/AKT pathway, promoting the expression of PTEN and suppressing the inflammatory response, this results in a favoring the M2 profile. Metformin, medication of choice for the treatment of type 2 diabetes has good anti-hyperglycemic effectiveness, but it is interesting also in wound healing approaches for having anti-inflammatory effects by regulating the AMPK/mTOR signaling pathway to inhibit the NLRP3 inflammasome activation and favoring M2 polarization. In this way, through the analysis of the potential behaviors of these key molecules, other biological processes could be activated as well.

## 5. Conclusions 

Studies have shown that specific combinations of hydrogels and bioactive molecules can regulate either innate or adaptive immune cell responses at various stages of wound healing. Investigating the interactions between immune cells and various types of natural/ synthetic hydrogels and bioactive compounds leads to critical mechanistic insights. However, the clinical translation of these structures requires more analysis on in vivo responses. Moreover, hydrogels can be designed for immunomodulatory therapy of chronic wounds via the delivery of bioactive molecules, including immunomodulatory components, or they can themselves intrinsically regulate either the innate or adaptive immune cell response (Figure 1).

One of the most promising treatments is focused on macrophages, as they are involved in key wound healing phases. In the inflammation phase, macrophages act by releasing molecules that help the process; however, once this process is finished, another spectrum of macrophages appears that release endless molecules to help resolve the inflammation. Consequently, the stimulation of the proliferation of other types of cells is required to promote wound healing.

The level of inflammation in wounds is dynamic, and the phenotype of macrophages varies according to the wound microenvironment. Macrophages display different phenotypes to perform various roles during the wound healing process. They exhibit a proinflammatory M1 phenotype in the early inflammatory stages and an anti-inflammatory M2 phenotype in the repair stages. A phenotypic continuum may exist during the process, with some cells sharing the phenotypic characteristics of the M1 and M2 macrophages. The phenotypic regulation of macrophages is a sophisticated process. Insufficient M1 macrophages in the early stages may lead to severe infection or delayed wound healing, whereas excessive M2 macrophages in the later stages may result in scar formation.

There are different strategies that promote the polarization of M2 and/or inhibition of M1 discussed in this manuscript, which shows the potential synergy of polymers/bioactive compounds as immunomodulators. This synergy of treatments where a biomaterial with favorable properties is proposed in terms of its actions toward macrophages, in combination with immunomodulatory compounds with the same properties, could shorten the wound healing times.

After conducting a survey of the latest reported polymers/bioactive compounds with wound healing activity promoted through immunomodulation, specifically with M1–M2 transformation effect, we propose four alternative combinations not previously reported that could accelerate the wound closure times: hyaluronic acid/melatonin and quercetin, collagen/metformin and quercetin, sulfobetaine methacrylate/metformin and melatonin, and sulfated chitosan/metformin and melatonin.

The current dressings lack the ability to precisely modulate the phenotype of macrophages to achieve ideal results. In addition, few studies on hydrogels have uncovered the molecular mechanisms of macrophage polarization, which is of great significance for the precise regulation of macrophage activity in wound healing. Therefore, while clearly more research is needed to solve this major issue, we propose these four functional hydrogel–immunomodulator combinations as promising biomaterials that could help in healing skin pathologies such as the ones suffered by diabetic patients.

## Figures and Tables

**Table 1:** Immunomodulator promoters of macrophage modulation in wound healing.

Immunomodulation Strategy	Pathway Alteration in Macrophage Phenotype	Effects on Wound Healing	Reference
ENDOGENOUS M1 ATTENUATION			
Quercetin	Inhibits the activation of TLR4/MyD88 signal transduction pathway, leading to the down-regulation of the activity of NF-kB and IRF-5, thus inhibiting the polarization of macrophages to the M1 phenotype, reducing the synthesis and release of inflammatory factors.	Reduce the infiltration of inflammatory cells. Increase fibroblast activity and collagen deposition. Promote angiogenesis.	[19,71]
ENDOGENOUS MACROPHAGE MODULATION/M2 PROMOTION			
IL-33	The binding of IL-33 to cells that express ST2 results in the activation of NF-kB and MAP kinases.	Accelerates re-epithelialization. Increases the proliferation of fibroblasts and ECM deposition.	[22,34]
HG plus insulin	Both the PI3K-Akt-Rac1 and PPAR-γ signaling pathways. Activates Akt-Rac-1 signaling.	Can decrease neutrophil infiltration. Accelerates vessel maturation.	[23]
Metformin	Regulates the AMPK/mTOR signaling pathway to inhibit NLRP3 inflammasome activation.	Improves angiogenesis. Inhibits the expression of pro-inflammatory cytokines. Accelerates collagen deposition. Re-vascularization, fibroblast regeneration and myofibroblast differentiation.	[64,66]
Melatonin	Upregulates the expression of PTEN, inhibiting the phosphorylation of AKT.	Facilitates angiogenesis and collagen synthesis. Suppresses the pro-inflammatory factors. Promotes the anti-inflammatory factor IL-10, along with increasing the relative expression of IL-10 and Arg-1.	[55,61]
Rapamycin	Reduces the NLRP3 inflammasome activation by inhibiting mTOR phosphorylation and NF-κB activation.	Enhances autophagy. Wound closure. Reduces the activation of the inflammatory cascade.	[70]

**Table 2:** Polymer promoters of macrophage modulation in wound healing.

Polymer	Type of Biomaterial	Main Alterations of Macrophages Phenotype	Effects on Wound Healing	References
Thioether grafted hyaluronic acid	Electro-spun nanofibers	Promotes the transformation of macrophages from a pro-inflammatory M1 to a reparative M2 phenotype.	Accelerates the healing phase transition from inflammation to proliferation and remodeling.	[29,80]
Sulfated chitosan (SCS)-doped collagen type I (Col I/SCS).	Porous hydrogel scaffolds	Reduces the polarization of M1-like macrophages.	Increases collagen deposition, re-epithelialization and neovascularization. Reduces the production of pro-inflammatory interleukin (IL)-6 and increases the production of anti-inflammatory cytokines, including IL-4 and transforming growth factor-beta 1 (TGF-β1).	[25]
Equine pericardial collagen matrix	Wound dressing	Change in macrophage polarization toward the M2 phenotype.	Accelerates wound re-epithelialization. Increases collagen deposition and maturation.	[25,30,83]
Sulfated poly (sulfobetaine methacrylate)	Wound dressing hydrogel	The polarization of macrophages from M1 to M2 through enhanced anti-inflammatory proteins.	Facilitates cell proliferation, granulation formation, collagen aggregation, chondrogenic ECM deposition, and neovascularization.	[84]

**Table 3:** The suggested combinations of polymers and immunomodulators that could work synergistically to promote wound healing and their potential impact on M1 and M2 profiles.

		Biomaterials			
		Equine Pericardial Collagen [25]	Hyaluronic Acid [31]	Sulfated Chitosan [26]	Sulfobetaine Methacrylate [78]
Immunomodulator	Wound Closure Period (Days)	13–15	13–15	16–18	19–21
Metformin [63]	10–12	M1 M2++	M1 M2++	M1 M2++	M1 M2++
Melatonin [56]	13–15	M1− M2++	M1−− M2++	M1− M2++	M1− M2++
Quercetin [54]	13–15	M1− M2++	M1−− M2++	M1− M2++	M1− M2++
IL-33 [55]	16–18	M1 M2++	M1− M2++	M1 M2++	M1 M2++
High glucose pus insulin [24]	ND	M1 M2++	M1− M2++	M1 M2++	M1 M2++
Rapamycin [70]	19–21	M1 M2++	M1− M2++	M1 M2++	M1 M2++

The notation "M1−" is used to indicate the ability of a material or compound to inhibit macrophage polarization toward the M1 phenotype. The notation "M2+" is used to indicate the ability of a material or compound to promote polarization of macrophages towards the M2 phenotype. While "M1−−" indicates the additive effect of a material and a compound having the ability to inhibit macrophages toward the M1 phenotype; and "M2++" indicates the additive effect of a mate-rial and a compound having the ability to promote macrophages toward the M2 phenotype.
